# Non-encapsulated *Streptococcus pneumoniae*, vaccination as a measure to interfere with horizontal gene transfer

**DOI:** 10.1080/21505594.2017.1309492

**Published:** 2017-03-22

**Authors:** J. D. Langereis, M. I. de Jonge

**Affiliations:** Laboratory of Pediatric Infectious Diseases, Radboud Center for Infectious Diseases, Radboud University Medical Center, Nijmegen, the Netherlands

**Keywords:** antibiotic resistance, non-encapsulated pneumococci, pneumococcal disease, vaccination

The polysaccharide capsule is the most important virulence factor of *Streptococcus pneumoniae*, however it is neither a requirement for persistent carriage nor for transmission. It has been postulated that reduced or complete lack of capsule expression facilitates colonization of the nasopharynx as a result of enhanced binding to epithelial cells.^1^ On the other hand, the large investment of energy in the synthesis of a polysaccharide capsule protect the pneumococci against complement mediated killing.^2^ Furthermore, a capsule might prevent agglutination in a mucus rich environment avoiding clearance from the nasopharyngeal cavity.^3^

An important group of pneumococci is serologically nontypeable (NT) due to the lack of a capsule, therefore called non-encapsulated *Streptococcus pneumoniae* (NESp). They are divided in 2 groups: group I, which contains a conventional but defective *cps* locus as a consequence of different types of mutations causing capsule gene inactivation and group II, which completely lacks a *cps* locus.^4^ The latter group consists of 3 ‘null capsule clades’ (NCC), classified based on the presence of 3 genes *pspK* (or *nspA*), *aliC* (or aliB-like ORF1) and *aliD* (or aliB-like ORF2): NCC1 (*pspK*^+^, *aliC*^−^, *aliD*^−^), NCC2 (*pspK*^−^, *aliC*^+^, *aliD*^+^), NCC3 (*pspK*^−^, *aliC*^−^, *aliD*^+^). The *pspK, aliC* and *aliD* genes have replaced the capsule locus, and recombination of flanking homologous genes (*dexB* and *aliA*) has the potential to transfer this gene cluster between encapsulated and non-encapsulated strains. It has been shown that group II NESp can colonize mouse nasopharynges as well as capsulated pneumococci, which implies that they have been able to compensate for the lack of a capsule enabling persistent carriage.^4^

The current pneumococcal vaccines are made of purified conjugated (10 or 13 valent) or non-conjugated (23 valent) polysaccharide capsules, which evidently do not protect against NESp. Vaccination with a limited number of serotypes leads to an increased pressure on a specific subset of pneumococci that opens an environmental niche that NESp strains are able to exploit.^5^ This might explain why an increase in NESp prevalence has been observed following the introduction of pneumococcal conjugate vaccines.^6^ Asymptomatic carriage rates of NESp have been measured, ranging from 4% to 19%.^7, 8^ Generally, NESp cause mainly non-invasive pneumococcal diseases such as otitis media and infectious conjunctivitis. In rare cases, infection with NESp can also lead to invasive pneumococcal disease, although mainly in immunodeficient patients. Nevertheless, the total number of clinical cases caused by NESp might be underestimated since serotyping is not routinely performed.^5^

A potentially more serious concern is the role of NESp in the spread of antibiotic resistance and virulence genes. The pneumococcus exploits different mechanisms to mediate horizontal gene transfer and recombination, including transformation, the use of mobile elements and transducing phages.^9^ Despite the great advantages of having a capsule, providing resistance to clearance during colonization, it strongly hampers DNA uptake limiting the capability of transformation-mediated adaptation. *In vitro* studies have shown that capsule-negative mutants acquire genes through recombination at a higher frequency than the isogenic encapsulated strains.^1, 10^ In the largest pneumococcal sequencing study thus far, with more than 3000 isolates, it was found that NESp showed the highest frequencies of receipt and donation of recombined DNA fragments, confirming that they are a potential major reservoir of genetic diversity for the wider population. The model proposed by Andam and Hanage explains how frequent loss and subsequent gain of capsule loci contribute to the observed variation in recombination rates and antibiotic resistance among pneumococcal lineages.^9^ It is proposed to start with random single nucleotide polymorphisms, deletions or transposon insertions leading to a group I NESp with increased capabilities of acquiring exogenous DNA, including virulence or antibiotic resistance genes. However, the sudden loss of capsule will make them more vulnerable for clearance, which is therefore likely a transient condition. The strain will either quickly regain its original or another capsule, or acquire the *pspK, aliC* and/or *aliD* genes to become a group II NESp, which is much better adapted than group I NESp allowing prolonged uptake of exogenous DNA. Subsequently, these group II NESp strains might at some point switch to an encapsulated state again. This cycle of encapsulation and un-encapsulation is thought to play an important role in the pneumococcal population dynamics and the spread of antibiotic resistance and virulence genes. This might explains why a significant proportion of NESp is resistant against one or multiple classes of antibiotics, as recently reviewed by.^5^

Jang *et al.* studied one of the most important proteins involved in capsule-independent persistence of carriage, the pneumococcal surface protein Korea (PspK), as potential vaccine antigen.^11^ The *pspK* gene is widely spread in NESp group II strains in Asia.^12^ Mature PspK, an LPxTG-anchored protein, consists of an undefined domain (UD), an R3 domain, showing homology with the R1 domain of PspC and both containing α-helical structures and a more conserved part containing stretches of 7 amino acid repeats. Deletion of the *pspK* gene led to reduced adherence but increased invasion into lung epithelial cells. PspK-mediated interaction with epithelial cells was dependent on a specific interaction between the R3 domain and Annexin-2, a membrane protein involved in actin-driven cellular uptake. Jang *et al.* showed furthermore that vaccination with R3 induced protection, showing the strongest effects measured after intranasal immunization.^11^ Despite the variability of PspK, antibodies raised against this protein cross-reacted with 79% of the encapsulated strains not containing PspK. This cross-reactivity is likely due to recognition of domains of distantly related proteins, such as PspC and PspA, therefore much higher cross-reactivity is expected with PspK containing NESp. Although the results are suggestive of an antibody-mediated mechanism of protection, it cannot be excluded that Th17 responses are induced, as has been shown to be essential for PspA-mediated protection.^13^

In conclusion, the study of Jang *et al.* shows the potential of PspK as vaccinate candidate,^11^ thereby also confirming earlier work from Keller *et al.* (2015).^14^ Whether the other group II NESp-specific proteins AliC and AliD are protective antigens remains to be studied. It is tempting to speculate that vaccination with proteins essential to compensate for the lack of a capsule such as PspK, AliC and AliD will result in strong reduction of colonization disrupting the chain of horizontal gene transfer and recombination. This might ultimately lead to the prevention of the emergence of capsule switching and antibiotic resistance.
